# Confidence-based Somatic Mutation Evaluation and Prioritization

**DOI:** 10.1371/journal.pcbi.1002714

**Published:** 2012-09-27

**Authors:** Martin Löwer, Bernhard Y. Renard, Jos de Graaf, Meike Wagner, Claudia Paret, Christoph Kneip, Özlem Türeci, Mustafa Diken, Cedrik Britten, Sebastian Kreiter, Michael Koslowski, John C. Castle, Ugur Sahin

**Affiliations:** 1 TRON - Translational Oncology at the Johannes Gutenberg University of Mainz Medicine, Mainz, Germany; 2 Research Group Bioinformatics (NG 4), Robert Koch-Institut, Berlin, Germany; 3 Department of Internal Medicine III, Division of Translational and Experimental Oncology, University Medical Center, Johannes Gutenberg University, Mainz, Germany; 4 Theracode GmbH, Mainz, Germany; 5 Ganymed Pharmaceuticals AG, Mainz, Germany; 6 Ribological GmbH, Mainz, Germany; Accelrys, United States of America

## Abstract

Next generation sequencing (NGS) has enabled high throughput discovery of somatic mutations. Detection depends on experimental design, lab platforms, parameters and analysis algorithms. However, NGS-based somatic mutation detection is prone to erroneous calls, with reported validation rates near 54% and congruence between algorithms less than 50%. Here, we developed an algorithm to assign a single statistic, a false discovery rate (FDR), to each somatic mutation identified by NGS. This FDR confidence value accurately discriminates true mutations from erroneous calls. Using sequencing data generated from triplicate exome profiling of C57BL/6 mice and B16-F10 melanoma cells, we used the existing algorithms GATK, SAMtools and SomaticSNiPer to identify somatic mutations. For each identified mutation, our algorithm assigned an FDR. We selected 139 mutations for validation, including 50 somatic mutations assigned a low FDR (high confidence) and 44 mutations assigned a high FDR (low confidence). All of the high confidence somatic mutations validated (50 of 50), none of the 44 low confidence somatic mutations validated, and 15 of 45 mutations with an intermediate FDR validated. Furthermore, the assignment of a single FDR to individual mutations enables statistical comparisons of lab and computation methodologies, including ROC curves and AUC metrics. Using the HiSeq 2000, single end 50 nt reads from replicates generate the highest confidence somatic mutation call set.

## Author Summary

Next generation sequencing (NGS) has enabled unbiased, high throughput discovery of genetic variations and somatic mutations. However, the NGS platform is still prone to errors resulting in inaccurate mutation calls. A statistical measure of the confidence of putative mutation calls would enable researchers to prioritize and select mutations in a robust manner. Here we present our development of a confidence score for mutations calls and apply the method to the identification of somatic mutations in B16 melanoma. We use NGS exome resequencing to profile triplicates of both the reference C57BL/6 mice and the B16-F10 melanoma cells. These replicate data allow us to formulate the false discovery rate of somatic mutations as a statistical quantity. Using this method, we show that 50 of 50 high confidence mutation calls are correct while 0 of 44 low confidence mutations are correct, demonstrating that the method is able to correctly rank mutation calls.

## Introduction

Next generation sequencing (NGS) has revolutionized our ability to determine genomes and compare, for example, tumor to normal cells to identify somatic mutations. However, the platform is not error free and various experimental and algorithmic factors contribute to the false positive rate when identifying somatic mutations [1]. Indeed, recent studies report validation rates of 54% [2]. Error sources include PCR artifacts, biases in priming [3], [4] and targeted enrichment [5], sequence effects [6], base calling causing sequence errors [7], variations in coverage, and uncertainties in read alignments [8], such as around insertions and deletions (indels) [9]. Reflecting the rapid development of bench and computational methods, algorithms to identify somatic mutations from NGS data are still evolving rapidly. Remarkably, the congruence of identified mutations between current algorithms is less than 50% (below).

Given the large discrepancies, one is left wondering which mutations to select, such as for clinical decision making or ranking for follow-up experiments. Ideal would be a statistical value, such as a p-value, indicating the confidence of each mutation call. Error sources have been addressed by examining bulk sets of mutations, such as computational methods to measure the expected amount of false positive mutation calls utilizing the transition/transversion ratio of a set of variations [10], [11], machine learning [12] and inheritance errors when working with family genomes [13] or pooled samples [14], [15]. Druley *et al.* [13] optimized variation calls using short plasmid sequence fragments for optimization. The accuracy of calling germline variations, i.e. single nucleotide polymorphisms (SNPs), has been addressed by validating SNPs using other techniques such as genotyping microarrays [15]. Thus, these methods enable a comparison of methods to identify and characterize error sources, but they do not assign a ranking score to individual mutation.

Several NGS mutation identification algorithms do output multiple parameters for each mutation call, such as coverage, genotype quality and consensus quality. However, it is not clear if and how to interpret these metrics with regards to whether a mutation call is correct. Furthermore, multiple parameters are generated for each mutation call and thus one simply cannot rank or prioritize mutations using the values. Instead, researchers often rely on personal experience and arbitrary filtering thresholds to select mutations. In summary, a) there is a low level of congruence between somatic mutations identified by different algorithms and sequencing platforms and b) no method to assign a single accuracy estimate to individual mutations.

Here, we develop a methodology to assign a confidence value - a false discovery rate (FDR) - to individual identified mutations. This algorithm does not identify mutations but rather estimates the accuracy of each mutation. The method is applicable both to the selection and prioritization of mutations and to the development of algorithms and methods. Using Illumina HiSeq reads and the algorithms GATK, SAMtools and SomaticSNiPer, we identified 4,078 somatic mutations in B16 melanoma cells. We assigned a FDR to each mutation and show that 50 of 50 mutations with low FDR (high confidence) validated while 0 of 44 with high FDR (low confidence) validated.

## Results

Somatic mutation discovery involves the determination and comparison of two genomes: the “normal” germline genome from non-cancerous cells and the tumor genome. If one, however, sequences one sample multiple times, such as the normal genome, and then compares the replicates, one should identify no differences. Thus, any mutation detected in this “same versus same comparison” is a false positive. These can be generated during sample extraction, sample preparation, amplification and library construction, NGS sequencing and data analysis.

**Figure 1:**
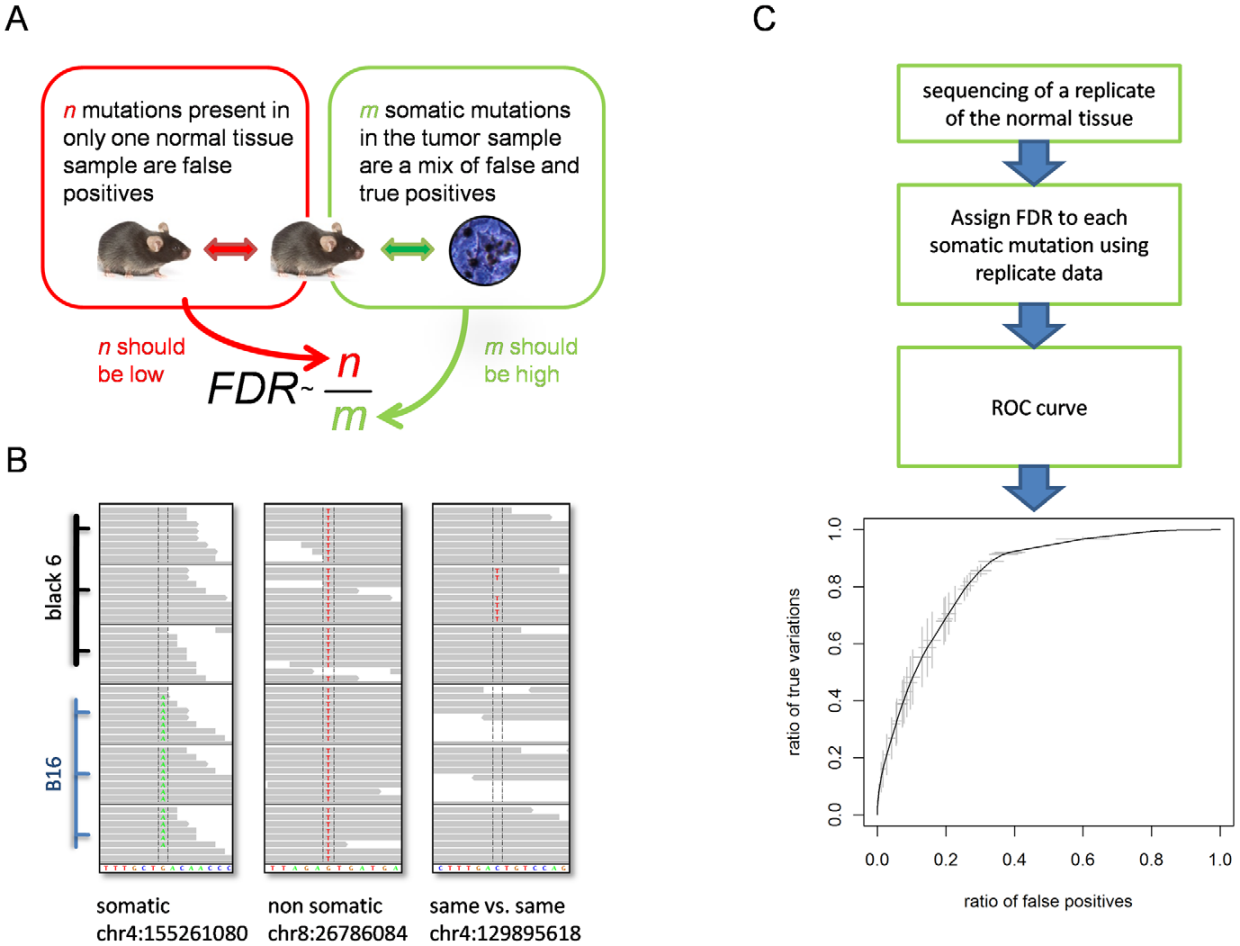
Schematic overview of FDR calculation method. **A** Concept of FDR calculation which relies on the availability of a normal tissue replication experiment. **B** Examples of single nucleotide variations found: A somatic mutation found in all three B16 samples (left), a non-somatic mutation found in all B16 and black6 samples (middle) and a mutation found in only one black6 sample (right); this last variation would cause a raise in the FDR for all somatic mutations with a comparable or worse quality. **C** Process to generate FDRs for a set of somatic mutations and visualize the results. The FDR distribution is visualized as an average estimated ROC curve with the grey bars giving the 95% confidence interval for the mean in both dimensions at uniformly sampled positions. The mean was obtained from the distribution of estimated ROC curves of the FDRs for all possible 18 combinations of reference data sets (see text).

To determine the false discovery rate (FDR) for each somatic mutation detected in a tumor sample relative to a normal sample (“tumor versus normal comparison”), we first define and assign a quality score Q to each identified mutation. Then, we count the number of “same versus same” mutations (false positives) at the same or greater quality score (Figure 1a) and convert this number of false positives into a FDR. In summary, the use of a single quality score and a “same versus same” mutation profile allow us to determine the FDR as a function of Q and assign an FDR to each mutation.

Figure 1b shows examples of variations found in sequences reads from B16F10 (B16) melanoma cells and the reference C57BL/6 (black6) mice. The variations include a somatic mutation found only in the tumor cells (left), a SNP found in both the black6 mice and B16 cells (middle) and probable false positive (right). We associate each mutation with a single quality score, the FDR, which defines the likelihood that the mutation call is inaccurate. Using the FDR, we plot receiver operating characteristic (ROC) curves and determine the corresponding area under curve (AUC) metric to compare the performance of different protocols and algorithms (Figure 1c).

### Mutation discovery

To discover mutations, DNA from tail tissue of three black6 mice, all litter mates, and DNA from three B16 melanoma samples, was extracted and exon-encoding sequences were captured, resulting in six samples. RNA was extracted from B16 cells in triplicate. Single end 50 nt (1×50 nt) and paired end 100 nt (2×100 nt) reads were generated on an Illumina HiSeq 2000 (Supplementary Table S1 in Text S1). Each sample was sequenced on an individual lane, resulting in an average of 104 million reads per lane. DNA reads were aligned to the mouse reference genome using the Burrows-Wheeler Alignment Tool (bwa) [16] and RNA reads were aligned with bowtie [17]. Using the 1×50 nt reads, 97% of the targeted nucleotides were covered at least once, the mean/median targeted nucleotide coverage was 38×/30× and 70–73% of target nucleotides had 20× or higher coverage. Using the 2×100 nt reads, 98% of the targeted nucleotides were covered at least once, the mean/median targeted nucleotide coverage the was 165×/133× and 97% of target nucleotides had 20× coverage.

**Figure 2:**
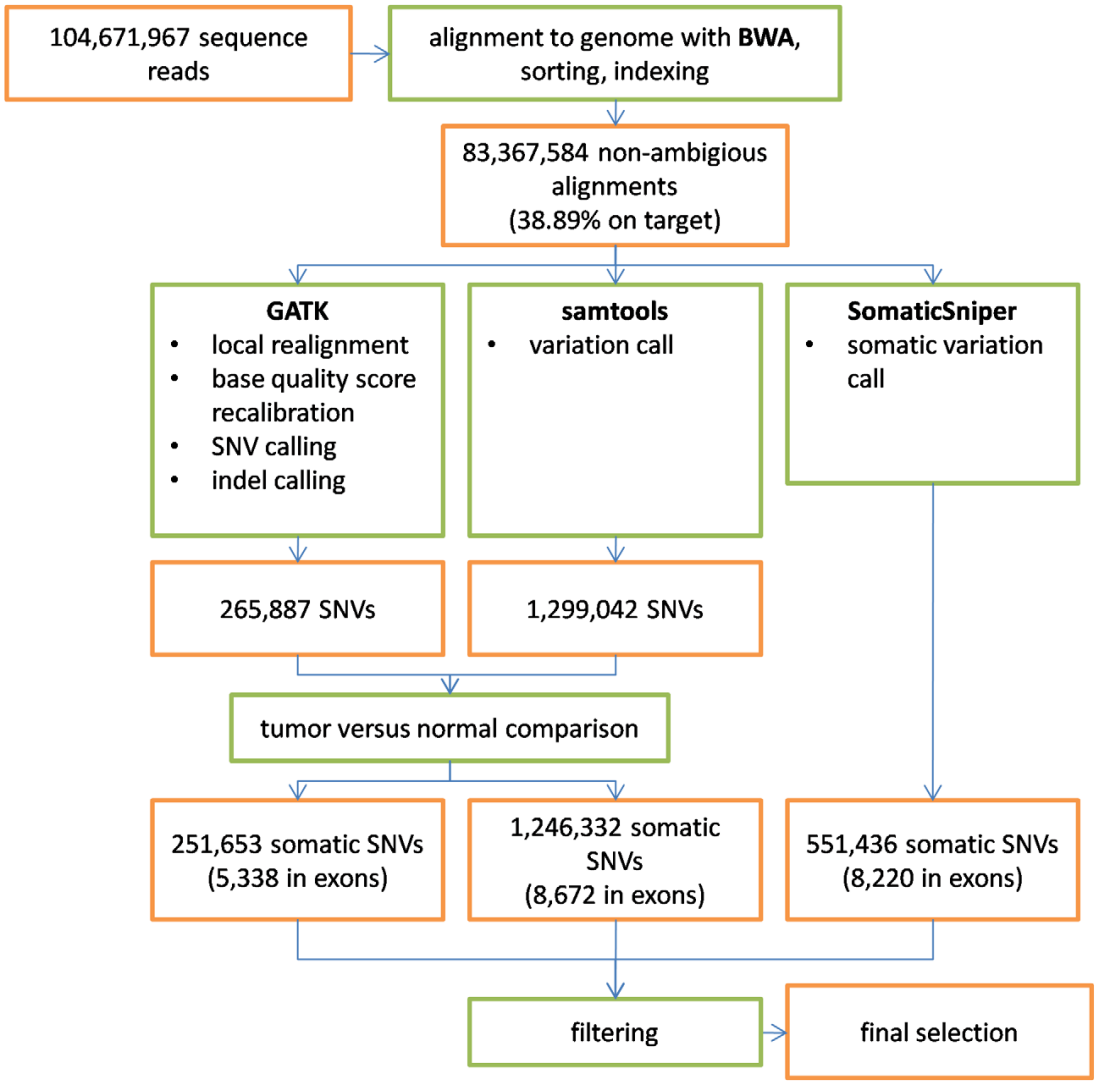
Overview of the process for finding somatic mutations in B16. Numbers for the individual steps are given as an example for one B16 sample, compared to one black6 sample. “Exons” refers to the exon coordinates defined by all protein coding RefSeq transcripts.

**Figure 3:**
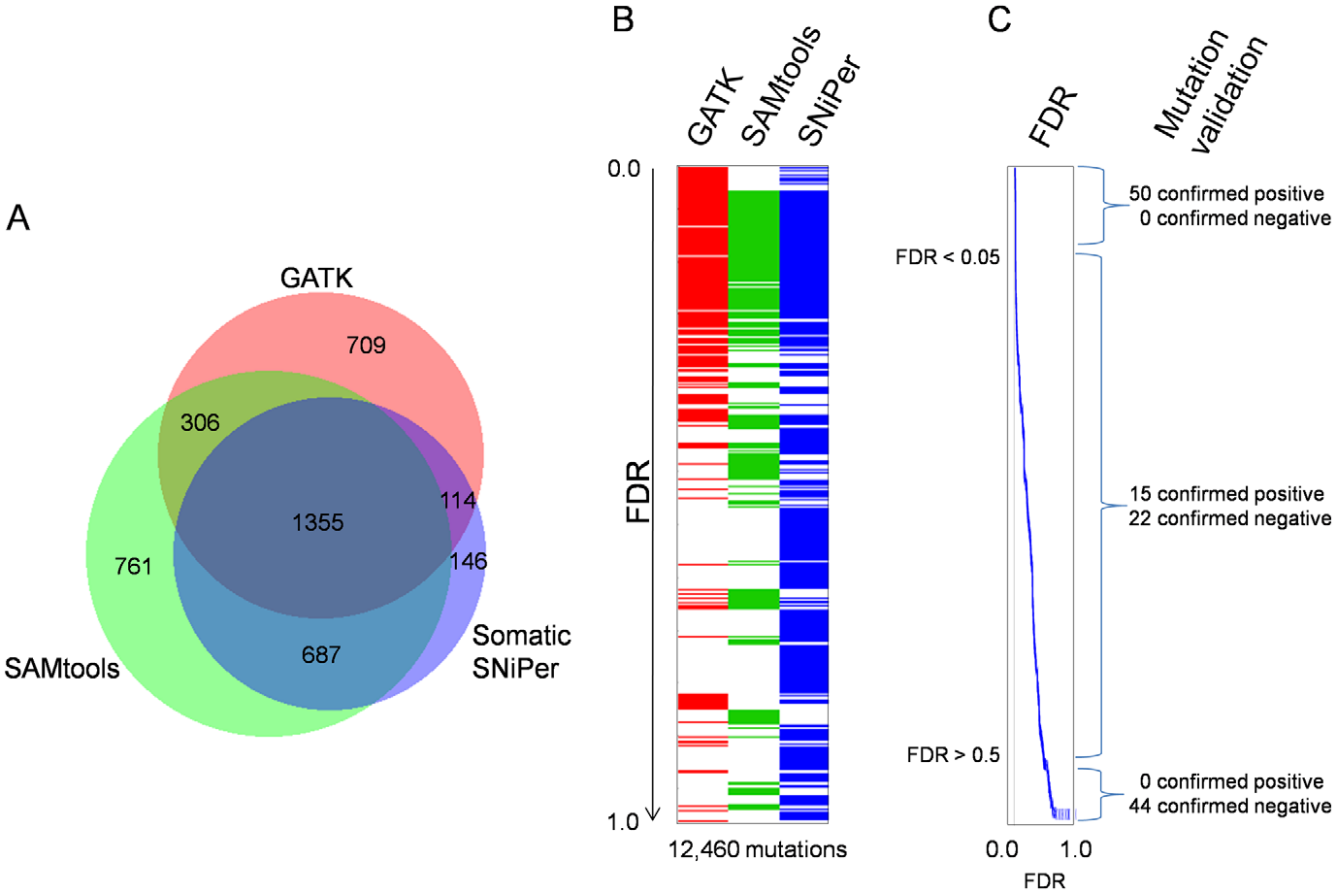
Process of selection of mutations for validation. **A** The Venn diagram shows the numbers of somatic variations in protein coding exons, found by the individual, two or all three software tools, respectively. The numbers were calculated after the recommended filtering procedures (see Methods section) and represent the consensus of all three samples. **B** List of unfiltered somatic mutations found in the consensus of all three samples, sorted by FDR (low to high from top to bottom). Each row represents a predicted mutation and it is indicated which program did the prediction. **C** For each mutation a FDR can be calculated, which is used for prioritization of the validation experiment.

Somatic mutations were independently identified using the software packages SAMtools [18], GATK [11] and SomaticSNiPer [19] (Figure 2) by comparing the single nucleotide variations found in B16 samples to the corresponding loci in the black6 samples (B16 cells were originally derived from a black6 mouse). The potential mutations were filtered according to recommendations from the respective software tools (SAMtools and GATK) or by selecting an appropriate threshold for the somatic score of SomaticSNiPer (Methods). Considering only those mutations found in all tumor-normal pairings, the union of B16 somatic mutations identified by the three algorithms was 4,078 (Figure 3a). However, substantial differences between the sets of mutations identified by each program exist, even when considering those mutations found in all tumor-normal pairings (Figure 3a). While 1,355 mutations are identified by all three programs (33% of 4,078), the agreement between results is low. Of the 2,484 mutations identified by GATK, only 1,661 (67%) are identified by SAMtools and 1,469 (60%) are identified by SomaticSNiPer. Of the 3,109 mutations identified by SAMtools, only 53% and 66% are identified by GATK and SomaticSNiPer, respectively. Of the 2,302 mutation identified by SomaticSNiPer, only 64% and 89% are identified by GATK and SAMtools, respectively. The number of 1,355 mutations identified by all three algorithms reflects only 55% (GATK), 44% (SAMtools) and 59% (SomaticSNiPer) of the mutations found by the individual programs, respectively.

### Mutations can be assigned a FDR confidence value

We want to assign each somatic mutation a single quality score Q that could be used to rank mutations based on confidence. However, it is not straightforward to assign a single value since most mutation detection algorithms output multiple scores, each reflecting a different quality aspect. Thus, we generated a random forest classifier [20] that combines multiple scores, resulting in a single quality score Q (Methods). All identified somatic mutations, whether from the “same versus same” or “tumor versus normal” comparison, thus are assigned a single value predicting accuracy. Note that the classifier training needs to be performed separately for each program, due to the differences in the set of scores which are returned by the individual programs.

After defining a relevant quality score, we sought to re-define the score into a statistically relevant false discovery rate (FDR). We determined, at each Q value, the number of mutations with a better Q score in the “same versus same” and the number of mutations with a better Q score in the “tumor versus normal” pair. For a given mutation with quality score Q detected in the “tumor versus normal” comparison, we estimate the false discovery rate by computing the ratio of “same versus same” mutations with a score of Q or better to the overall number of mutations found in the tumor comparison with a score of Q or better.

A potential bias in comparing methods is differential coverage; we thus normalize the false discovery rate for the number of bases covered by NGS reads in each sample:
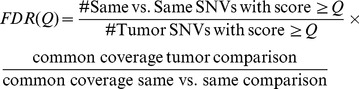



We calculate the common coverage by counting all bases of the reference genome which are covered by data of the tumor and normal sample or by both “same versus same” samples, respectively. After assigning our FDR to each mutation, the FDR-sorted list of somatic mutations shows a clear preference of mutations found by three programs in the low FDR region (Figure 3b; see Supplementary Dataset S1 for a complete list). This observation fits to the naïve assumption that the consensus of multiple different algorithms is likely to be correct.

We identified 50 mutations with a low FDR (high confidence) for validation, including 41 with an FDR less than 0.05 (Figure 3c). All 50 were validated by a combination of Sanger resequencing and inspection of the B16 RNA-Seq sequence reads. Table 1 lists the ten somatic mutations with the best FDRs, all of which validated.

**Table 1:** Ten validated mutations with the lowest FDRs, selected out of a set of 2396 exonic variations which were found in duplicate in two B16 samples.

Chromosome	Position	Reference allele	Sample allele(s)	FDR
8	110078987	G	A/G	0.006
1	59540714	G	G/C	0.007
5	124854313	G	G/T	0.007
10	59352802	C	A/C	0.007
16	36919828	A	A/C	0.007
2	144078227	C	C/T	0.007
8	12834637	G	G/C	0.007
19	6121411	T	C/T	0.007
1	58533360	A	A/C	0.007
15	98478052	A	A/G	0.007

None of these mutations is present in dbSNP (version 128; genome assembly mm9).

**Figure 4:**
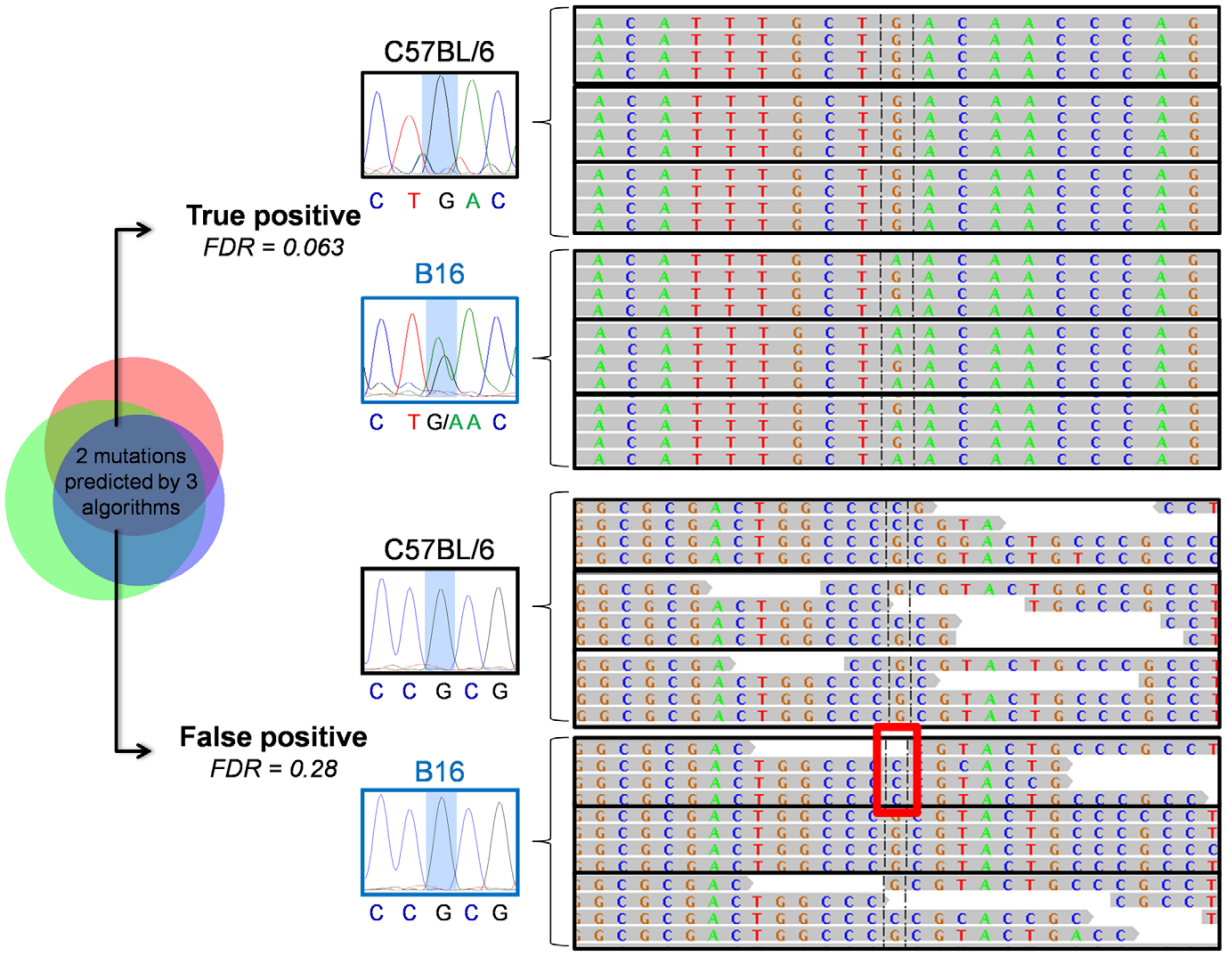
Genome browser screen shot for triplicate black6 and B16 samples and associated Sanger sequencing traces for the black6 and B16 DNA for both a true positive (chr4:155261079) and a false positive (chr4:151534480) mutation call. Both mutations are predicted by GATK, SomaticSNiPer and SAMtools. The mean coverage is 54 (true positive) and 10 (false positive), respectively. Only four reads are shown for visual clarity. The red box marks the sample, in which the three mutation callers wrongly detected a SNV.

We selected 44 mutations identified by at least one detection algorithm, present in only one B16 sample and assigned a high FDR (>0.5) by our algorithm (Figure 3c). In contrast to the low-FDR mutations, none of the 44 high FDR samples validated, neither by Sanger sequencing nor by inspection of the RNA alignments. 37 of those mutations were clear false positives (no mutation by Sanger or RNA-Seq) while the remaining seven loci neither yielded sequencing reactions nor were covered by RNA-Seq reads. Figure 4 shows representative mutations together with the Sanger sequencing traces. In the case of the false positive mutation, the three used programs identified this in black6 as sequencing error (and did not output a mutation at this locus), but failed in the single B16 case (marked with the red box). If a real experiment would have included only this single sample, it would have produced a false positive mutation call, despite using the consensus of three programs.

To test mutations with less extreme FDRs, we selected 45 somatic mutations, which were distributed evenly across the FDR spectrum from 0.1 to 0.6. Validation using both Sanger sequencing and inspection of the RNA-Seq reads resulted 15 positive (either Sanger sequencing or RNA-Seq reads), 22 negative validations (neither Sanger sequencing nor RNA-Seq reads) and 8 non-conclusive (failed sequencing reactions and no RNA-Seq coverage). See the Supplementary Dataset S2 for a detailed table showing the results of the validation of those 45 mutations.

**Figure 5:**
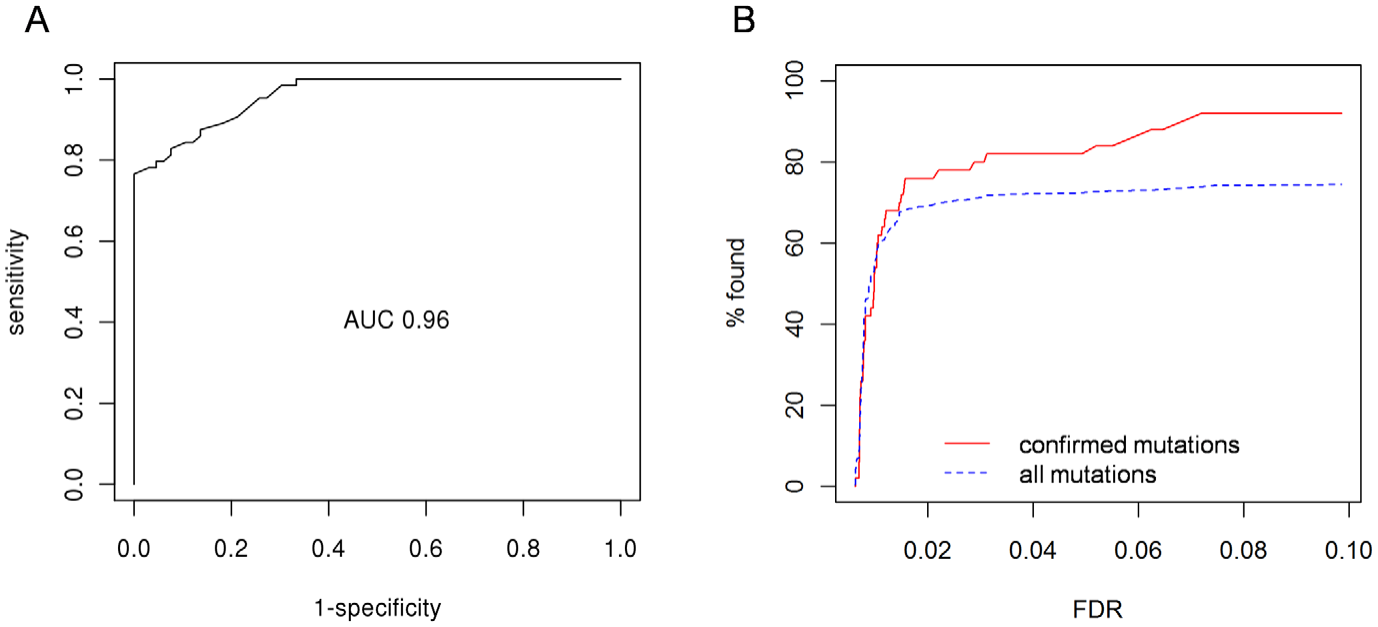
Results of mutation validation experiments. **A** ROC curve for all 131 mutations with a successful validation (either positive or negative). 1-FDR was used as the probability of a mutation being a true call. **B** Relative amount of variations found for a given FDR cutoff in a set of 2396 exonic variations which were found in duplicate in two B16 samples (see also Table 1), plotted separately for all variants in the dataset and the 50 validated high confidence mutations. For visual clarity only values of 0 to 0.1 FDR are shown.

We computed a receiver operating characteristic (ROC) curve for all 131 validated mutations (Figure 5a), resulting in an area under the curve (AUC) [21] of 0.96. As this analysis might be biased due to the relatively large set sizes of the high and low FDR mutations, we randomly sampled 10 mutations each, added the 37 validated mutations with the intermediate FDRs, calculated the ROC-AUC and repeated this 1000 times in order to get a more robust performance estimate. The resulting mean AUC is 0.797 (+−0.002). A systematic test of FDR thresholds ranging from zero to one with a step size of 0.05 implies that an optimal threshold for using the FDR as a binary classifier should be ≤0.2.

### ROC curves can be used to compare methods

ROC curves and the corresponding AUC are useful for comparing classifiers and visualizing their performance [21]. We extended this concept for evaluating the performance of experimental and computational procedures. However, plotting ROC graphs requires knowledge of all true and false positives (TP and FP) in a dataset, information which is usually not given and hard to establish for high throughput data (such as NGS data). Thus, we used the calculated FDRs to estimate the respective TP and FP rates and plot a ROC curve and calculate the AUC (Figure 1c). Figure 5b shows the ROC curve comparing the FDR versus the percent of 50 validated mutations and percent of total.

### Benchmarking of experimental settings

ROC curves and the associated AUC values can be compared across experiments, lab protocols, and algorithms. For the following comparisons, we used all somatic mutations found by any algorithm and in any tumor-normal pairing without applying any filter procedure. We considered only those mutations in target regions (exons).

First, we tested the influence of the reference “same versus same” data on the calculation of the FDRs. Using the triplicate black6 and B16 sequencing runs, we created 18 triplets (combinations of “black6 versus black6” and “black6 versus B16”) to use for calculating the FDR. When comparing the resulting FDR distributions for the sets of somatic mutations, the results are consistent when the reference data sets are exchanged (Figure 1c, Supplementary Figure S2 in Text S1). This suggests that the method is robust with regards to the choice of the reference “same versus same” dataset. Thus, a “same versus same” duplicate profiling needs only be done once for a given lab platform and the resultant FDR(Q) reference function can be re-used for future profiling.

Using our definition of a false discovery rate, we have established a generic framework for evaluating the influence of numerous experimental and algorithmic parameters on the resulting set of somatic mutations. We apply this framework to study the influence of software tools, coverage, paired end sequencing and the number of technical replicates on somatic mutation identification.

**Figure 6:**
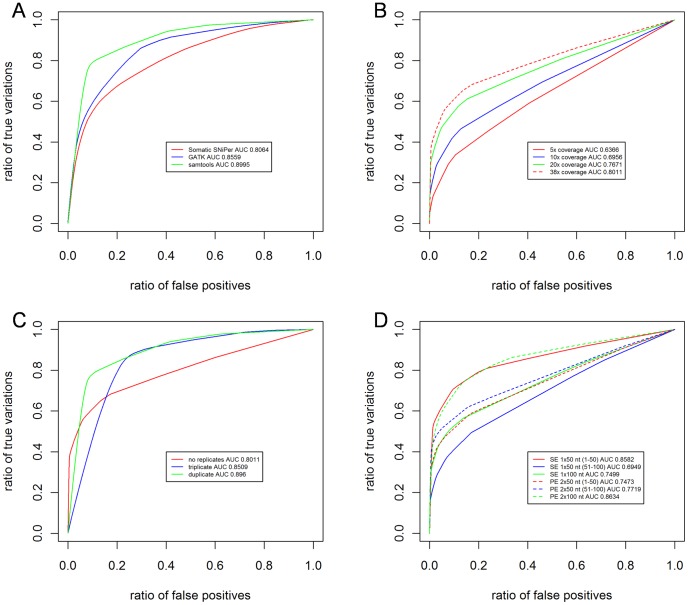
Comparison of different experimental settings and analysis procedures. **A** Estimated ROC curves for the comparison of the three different software tools (duplicates, 38× coverage). **B** Estimated ROC curves for the comparison of different average sequencing depths (SAMtools, no replication). 38× denotes the coverage obtained by the experiment, while other coverages were down sampled starting with this data. **C** Estimated ROC curves visualizing the effect of experiment replication (38× coverage, SAMtools). **D** Estimated ROC curves for different sequencing protocols (SAMtools, no replication). The curves were calculated using the results of the 2×100 nt library (**Note**: A complete display of the results can be found in Supplementary Figures S2 and S3 in Text S1. Also, unscaled versions of the plots are shown in Supplementary Figure S8 in Text S1, giving an impression of the individual set sizes).

First, the choice of the software tool has a clear impact on the identified somatic mutations (Figure 3). On the tested data, SAMtools produces the highest enrichment of true positive somatic mutations (Figure 6a). We note that each tool has different parameters and quality scores for mutation detection; we used the default settings as specified by the algorithm developers.

The impact of the coverage depth on whole genome SNV detection has been recently discussed [22]. For the B16 sequencing experiment, we sequenced each sample in an individual flowcell lane and achieved a target region mean base coverage of 38 fold across target nucleotides. In order to study the effect of the coverage on exon capture data, we down-sampled the number of aligned sequence reads for every 1×50 nt library to generate a mean coverage of 5, 10 and 20 fold, respectively, and then reapplied the mutation identification algorithms. As expected, a higher coverage results in a better (i.e. fewer false positives) somatic mutation set, although the improvement from the 20 fold coverage to 38 fold is marginal for the B16 cells (Figure 6b).

It is straightforward to simulate and rank other experimental settings using the available data and framework (Figures 6c and d). As we profiled each sample in triplicate, including three separate exon captures, we wanted to identify the impact of these replicates. Comparing duplicates to triplicates, triplicates do not offer a benefit compared to the duplicates (Figure 6c), while duplicates offer a clear improvement compared to a study without any replicates (indicated by the higher AUC). In terms of the ratio of somatic mutations at a FDR of 0.05 or less, we see enrichment from 24% for a run without replicates to 71% for duplicates and 86% for triplicates. These percentages correspond to 1441, 1549 and 1524 mutations, respectively. Using the intersection of triplicates removes more mutations with low FDRs than mutations with a high FDR, as indicated by the lower ROC AUC and the shift of the curve to the right (Supplementary Figure S7 in Text S1, Figure 6c): the specificity is slightly increased at the cost of a lower sensitivity, when assuming that removed low FDR mutations are true positives and the removed high FDR mutations are true negatives. This assumption is supported by our validation experiments, as true negative mutations are likely to get a high FDR (Figure 5a).

The 2×100 nt library was used to create 6 libraries: a 2×100 nt library; a 1×100 nt library; a 1×50 nt library using the 50 nucleotides at the 5′ end of the first read; a 1×50 nt library using the nucleotides 51 to 100 at the 3′ end of the first read; a 2×50 nt read using nucleotides 1 to 50 of both reads; and a 2×50 nt library using nucleotides 51 to 100 of both reads. These libraries were compared using the calculated FDRs of predicted mutations (Figure 6d). The 1×50 3′ library performed worst, as expected due to the increasing error rate at the 3′ end of sequence reads. Despite the much higher median coverage (63–65 vs. 32), the somatic mutations found using the 2×50 5′ and 1×100 nt libraries have a smaller AUC than the 1×50 nt library. This surprising effect is a result of high FDR mutations in regions with low coverage (Supplementary Text S1). Indeed, the sets of low FDR mutations are highly similar. Thus, while the different read lengths and types identify non-identical mutations, the assigned FDR is nevertheless able to segregate true and false positives (Supplementary Figure S3 in Text S1).

## Discussion

NGS is a revolutionary platform for detecting somatic mutations. However, the error rates are not insignificant, with different detection algorithms identifying mutations with less than 50% congruence. Other high throughput genomic profiling platforms have developed methods to assign confidence values to each call, such as p-values associated with differential expression calls from oligonucleotide microarray data. Similarly, we developed here a method to assign a confidence value (FDR) to each identified mutation.

From the set of mutations identified by the different algorithms, the FDR accurately ranks mutations based on likelihood of being correct. Indeed, we selected 50 high confidence mutations and all 50 validated; we selected 45 intermediate confidence mutations and 15 validated, 22 were not present and 8 inconclusive; we selected 44 low confidence mutations and none validated. Again, all 139 mutations were identified by at least one of the detection algorithms. Unlike a consensus or majority voting approach, the assigned FDR not only effectively segregates true and false positives but also provides both the likelihood that the mutation is true and a statistically ranking. Also, our method allows the adjustment for a desired sensitivity or specificity which enables the detection of more true mutations than a consensus or majority vote, which report only 50 or 52 of all 65 validated mutations.

We applied the method to a set of B16 melanoma cell experiments. However, the method is not restricted to these data. The only requirement is the availability of a “same versus same” reference dataset, meaning at least a single replicate of a non-tumorous sample should be performed for each new protocol. Our experiments indicate that the method is robust with regard to the choice of the replicate, so that a replicate is not necessarily required in every single experiment. Once done, the derived FDR(Q) function can be reused when the Q scores are comparable (i.e. when the same program for mutation discovery was used). Here, we profiled all samples in triplicate; nevertheless, the method produces FDRs for each mutation from single-run tumor and normal profiles (non-replicates) using the FDR(Q) function. We do show, however, that duplicates improve data quality.

Furthermore, the framework enables one to define best practice procedures for the discovery of somatic mutations. For cell lines, at least 20-fold coverage and a replicate achieve close to the optimum results. A 1×50 nt library resulting in approximately 100 million reads is a pragmatic choice to achieve this coverage.

The possibility of using a reference data set to rank the results of another experiment can also be exploited to e.g. score somatic mutations found in different normal tissues by similar methods. Here, one would expect relatively few true mutations, so an independent set of reference data will improve the resolution of the FDR calculations.

While we define the optimum as the lowest number of false positive mutation calls, this definition might not suffice for other experiments, such as for genome wide association studies. However, our method allows the evaluation of the sensitivity and specificity of a given mutation set and we show application of the framework to four specific questions. The method is by no means limited to these parameters, but can be applied to study the influence of all experimental or algorithmic parameters, e.g. the influence of the alignment software, the choice of a mutation metric or the choice of vendor for exome selection.

In summary, we have pioneered a statistical framework for the assignment of a false-discovery-rate to the detection of somatic mutations. This framework allows for a generic comparison of experimental and computational protocol steps on generated quasi ground truth data. Furthermore, it is applicable for the diagnostic or therapeutic target selection as it is able to distinguish true mutations from false positives.

## Methods

### Library capture and sequencing

Next-generation sequencing, DNA sequencing: Exome capture for DNA resequencing was performed using the Agilent Sure-Select solution-based capture assay [23], in this case designed to capture all known mouse exons.

3 µg purified genomic DNA was fragmented to 150–200 nt using a Covaris S2 ultrasound device. gDNA fragments were end repaired using T4 DNA polymerase, Klenow DNA polymerase and 5′ phosphorylated using T4 polynucleotide kinase. Blunt ended gDNA fragments were 3′ adenylated using Klenow fragment (3′ to 5′ exo minus). 3′ single T-overhang Illumina paired end adapters were ligated to the gDNA fragments using a 10∶1 molar ratio of adapter to genomic DNA insert using T4 DNA ligase. Adapter ligated gDNA fragments were enriched pre capture and flow cell specific sequences were added using Illumina PE PCR primers 1.0 and 2.0 and Herculase II polymerase (Agilent) using 4 PCR cycles.

500 ng of adapter ligated, PCR enriched gDNA fragments were hybridized to Agilent's SureSelect biotinylated mouse whole exome RNA library baits for 24 hrs at 65°C. Hybridized gDNA/RNA bait complexes where removed using streptavidin coated magnetic beads. gDNA/RNA bait complexes were washed and the RNA baits cleaved off during elution in SureSelect elution buffer leaving the captured adapter ligated, PCR enriched gDNA fragments. gDNA fragments were PCR amplified post capture using Herculase II DNA polymerase (Agilent) and SureSelect GA PCR Primers for 10 cycles. Cleanups were performed using 1.8× volume of AMPure XP magnetic beads (Agencourt). For quality controls we used Invitrogen's Qubit HS assay and fragment size was determined using Agilent's 2100 Bioanalyzer HS DNA assay. Exome enriched gDNA libraries were clustered on the cBot using Truseq SR cluster kit v2.5 using 7 pM and sequenced on the Illumina HiSeq2000 using Truseq SBS kit.

### Exome data analysis

Sequence reads were aligned using bwa (version 0.5.8c) [16] using default options to the reference mouse genome assembly mm9 [24]. Ambiguous reads – those reads mapping to multiple locations of the genome as provided by the bwa output - were removed (see Supplementary Dataset S3 for the alignment statistics). The remaining alignments were sorted, indexed and converted to a binary and compressed format (BAM) and the read quality scores converted from the Illumina standard phred+64 to standard Sanger quality scores using shell scripts.

For each sequencing lane, mutations were identified using three software programs: SAMtools pileup (version 0.1.8) [18], GATK (version 1.0.4418) [11] and SomaticSNiPer [19]. For SAMtools, the author-recommend options and filter criteria were used (http://sourceforge.net/apps/mediawiki/SAMtools/index.php?title=SAM_FAQ; accessed September 2011), including first round filtering, maximum coverage 200. For SAMtools second round filtering, the point mutation minimum quality was 30. For GATK mutation calling, we followed the author-designed best practice guidelines presented on the GATK user manual (http://www.broadinstitute.org/gsa/wiki/index.php?title=Best_Practice_Variant_Detection_with_the_GATK_v2&oldid=5207; accessed October 2010). For each sample a local realignment around indel sites followed by a base quality recalibration was performed. The Unified Genotyper module was applied to the resultant alignment data files. When needed, the known polymorphisms of the dbSNP [25] (version 128 for mm9) were supplied to the individual steps. The variant score recalibration step was omitted and replaced by the hard-filtering option. For both SAMtools and GATK, potential indels were filtered out of the results before further processing and a mutation was accepted as somatic if it is present in the data for B16 but not in the black6 sample. Additionally, as a post filter, for each potentially mutated locus we required non-zero coverage in the normal tissue. This is intended to sort out mutations which only look to be somatic because of a not covered locus in the black6 samples. For SomaticSNiPer mutation calling, the default options were used and only predicted mutations with a “somatic score” of 30 or more were considered further (see Supplementary Text S1 for a description of the cutoff selection). For all three programs, we removed all mutations located in repetitive sequences as defined by the RepeatMasker track of the UCSC Genome Browser [26] for the mouse genome assembly mm9.

### RNA-Seq

Barcoded mRNA-seq cDNA libraries were prepared from 5 ug of total RNA using a modified version of the Illumina mRNA-seq protocol. mRNA was isolated using SeramagOligo(dT) magnetic beads (Thermo Scientific). Isolated mRNA was fragmented using divalent cations and heat resulting in fragments ranging from 160–200 bp. Fragmented mRNA was converted to cDNA using random primers and SuperScriptII (Invitrogen) followed by second strand synthesis using DNA polymerase I and RNaseH. cDNA was end repaired using T4 DNA polymerase, Klenow DNA polymerase and 5′ phosphorylated using T4 polynucleotide kinase. Blunt ended cDNA fragments were 3′ adenylated using Klenow fragment (3′ to 5′ exo minus). 3′ single T-overhang Illumina multiplex specific adapters were ligated on the cDNA fragments using T4 DNA ligase. cDNA libraries were purified and size selected at 300 bp using the E-Gel 2% SizeSelect gel (Invitrogen). Enrichment, adding of Illumina six base index and flow cell specific sequences was done by PCR using Phusion DNA polymerase (Finnzymes). All cleanups were performed using 1.8× volume of Agencourt AMPure XP magnetic beads.

Barcoded RNA-seq libraries were clustered on the cBot using Truseq SR cluster kit v2.5 using 7 pM and sequenced on the Illumina HiSeq2000 using Truseq SBS kit.

The raw output data of the HiSeq was processed according to the Illumina standard protocol, including removal of low quality reads and demultiplexing. Sequence reads were then aligned to the reference genome sequence [24] using bowtie [17]. The alignment coordinates were compared to the exon coordinates of the RefSeq transcripts [27] and for each transcript the counts of overlapping alignments were recorded. Sequence reads not aligning to the genomic sequence were aligned to a database of all possible exon-exon junction sequences of the RefSeq transcripts [27]. The alignment coordinates were compared to RefSeq exon and junction coordinates, reads counted and normalized to RPKM (number of reads which map per nucleotide kilobase of transcript per million mapped reads [28]) for each transcript.

### Validation of SNVs

We selected SNVs for validation by Sanger re-sequencing and RNA. SNVs were identified which were predicted by all three programs, non-synonymous and found in transcripts having a minimum of 10 RPKM. Of these, we selected the 50 with the highest SNP quality scores as provided by the programs. As a negative control, 44 SNVs were selected which have a FDR of 0.5 or more, are present in only one cell line sample and are predicted by only one mutation calling program. 45 mutations with intermediate FDR levels were selected. Using DNA, the selected variants were validated by PCR amplification of the regions using 50 ng of DNA (see Supplementary Dataset S4 for the primer sequences and targeted loci), followed by Sanger sequencing (Eurofins MWG Operon, Ebersberg, Germany). The reactions were successful for 50, 32 and 37 loci of positive, negative and intermediate controls, respectively. Validation was also done by examination of the tumor RNA-Seq reads.

### Calculation of FDRs and machine learning

Random Forest Quality Score Computation: Commonly-used mutation calling algorithms ([11], [18], [19]) output multiple scores, which all are potentially influential for the quality of the mutation call. These include - but are not limited to - the quality of the base of interest as assigned by the instrument, the alignment quality and number of reads covering this position or a score for the difference between the two genomes compared at this position. For the computation of the false discovery rate we require an ordering of mutations, however this is not directly feasible for all mutations since we might have contradicting information from the various quality scores.

We use the following strategy to achieve a complete ordering. In a first step, we apply a very rigorous definition of superiority by assuming that a mutation has better quality than another if and only if it is superior in all categories. So a set of quality properties S = (s_1_,…,s_n_) is preferable to T = (t_1_,…,t_n_), denoted by S>T, if s_i_>t_i_ for all i = 1,…,n. We define an intermediate FDR (IFDR) as follows
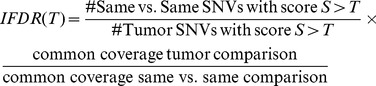



However, we regard the IFDR only as an intermediate step since in many closely related cases, no comparison is feasible and we are thus not benefitting from the vast amount of data available. Thus, we take advantage of the good generalization property of random forest regression [20] and train a random forest as implemented in R ([29], [30]).

For *m* input mutations with *n* quality properties each, the value range for each property was determined and up to *p* values were sampled with uniform spacing out of this range; when the set of values for a quality property was smaller than *p*, this set was used instead of the sampled set. Then each possible combination of sampled or selected quality values was created, which resulted in a maximum of *p^n^* data points in the *n*-dimensional quality space. A random sample of 1% of these points and the corresponding IFDR values were used as predictor and response, respectively, for the random forest training.

The resulting regression score is our generalized quality score Q; it can be regarded as a locally weighted combination of the individual quality scores. It allows direct, single value comparison of any two mutations and the computation of the actual false discovery rate:
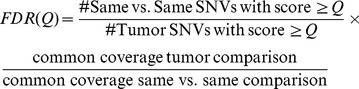



For the training of the random forest models used to create the results for this study, we calculate the sample IFDR on the somatic mutations of all samples before selecting the random 1% subset. This ensures the mapping of the whole available quality space to FDR values. We used the quality properties “SNP quality”, “coverage depth”, “consensus quality” and “RMS mapping quality” (SAMtools, *p* = 20); “SNP quality”, “coverage depth”, “Variant confidence/unfiltered depth” and “RMS mapping quality” (GATK, *p* = 20); or SNP quality”, “coverage depth”, “consensus quality”, “RMS mapping quality” and “somatic score” (SomaticSNiPer, *p* = 12), respectively. The different values of *p* ensure a set size of comparable magnitude.

To acquire the “same vs. same” and “same vs. different” data when calculating the FDRs for a given set of mutations, we use all variants generated by the different programs without any additional filtering.

Common coverage computation: The number of possible mutation calls can introduce a major bias in the definition of a false discovery rate. Only if we have the same number of possible locations for mutations to occur for our tumor comparison and for our “same vs. same” comparison, the number of called mutations is comparable and can serve as a basis for a false discovery rate computation. To correct for this potential bias, we use the common coverage ratio. As common coverage we define the number of bases with coverage of at least one in both samples which are used for the mutation calling. We compute the common coverage individually for the tumor comparison as well as for the “same vs. same” comparison.

### ROC estimation

The estimation of the ROC curves should satisfy the following criteria:

1. When all calculated FDRs are 0.5, one cannot use these rates to select true positive mutations. This should be reflected by a diagonal line from (0,0) to (1,1) in the ROC plot resulting in a ROC AUC of 0.5, which indicates a completely random prediction.
2. The normal calculation of ROC curves involves summing up the TP counts and FP counts, respectively, up to a given score threshold. Here, an individual mutation does not add one to the TP or FP count, but a fraction depending on the given FDR to both sums, respectively. Both fractions should add to one, then.

We start with two conditions; Eq. [1] is the definition of the FDR and Eq. [2] is needed to satisfy the criteria given above.
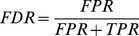
(1)

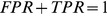
(2)
*FPR* and *TPR* are the needed false positive true positive ratios, respectively, for the given mutation, defining the corresponding point in ROC space. Eq. [1] and Eq. [2] can be rearranged to Eq. [3] and Eq. [4].
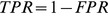
(3)

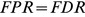
(4)


To obtain an estimated ROC curve, the mutations in the dataset are sorted by FDR and for each mutation a point is plotted at the cumulative TPR and FPR values up to this mutation, divided by the sum of all TPR and FPR values, respectively. The AUC is calculated by summing up the areas of all consecutive trapezoids between the curve and the x-axis.

The program is implemented in R and is available from http://tron-mainz.de/tron-facilities/computational-medicine/. The package allows convenient import and processing of variation calls in VCF files.

## Supporting Information

Dataset S1 12,460 somatic mutations found in triplicate samples.
(XLS)

Dataset S2 Validation results for mutations with an intermediate FDR.
(XLS)

Dataset S3 Alignment statistics for all samples.
(XLS)

Dataset S4 Primer sequences.
(XLS)

Text S1 Selection of a filtering threshold for SomaticSNiPer, discussion of paired end library results and additional figures (S1, S2, S3, S4, S5, S6, S7, S8) and tables (S1, S2, S3).
(PDF)
