# Supplementary material for: Confidence-based Somatic Mutation Evaluation and Prioritization
Source: PLoS Comput Biol. 2012 Sep 27;8(9):e1002714. doi: 10.1371/journal.pcbi.1002714 (PMC3459886; doi:10.1371/journal.pcbi.1002714)
Supplement: Text S1 — Selection of a filtering threshold for SomaticSNiPer, discussion of paired end library results and additional figures (S1, S2, S3, S4, S5, S6, S7, S8) and tables (S1, S2, S3). (PDF) [file pcbi.1002714.s005.pdf]

Supplementary Material

Confidence-based Somatic Mutation Evaluation and Prioritization

Martin Löwer[1,*], Bernhard Y. Renard[1,2,*], Jos de Graaf[1], Meike Wagner[1,3], Claudia Paret[1], Christoph Kneip[4], Özlem Türeci[5], Mustafa Diken[1], Cedrik Britten[6], Sebastian Kreiter[1], Michael Koslowski[1], John C. Castle[1,**], Ugur Sahin[1,**]

[1]TRON - Translational Oncology at the Johannes Gutenberg University of Mainz Medicine, Langenbeckstr. 1, Building 708, 55131 Mainz, Germany

[2] Research Group Bioinformatics (NG 4), Robert Koch-Institut, Nordufer 20, 13353 Berlin, Germany

[3] Department of Internal Medicine III, Division of Translational and Experimental Oncology,

University Medical Center, Johannes Gutenberg University, 55131 Mainz, Germany

[4] Theracode GmbH, Mainz, Germany

[5] Ganymed Pharmaceuticals AG, Mainz, Germany

[6] Ribological GmbH, Mainz, Germany

* Contributed equally

** Corresponding authors; both authors share last authorship

Contact:

Ugur Sahin (sahin@uni-mainz.de)

John C. Castle (John.Castle@TrOn-Mainz.DE)

TRON - Translational Oncology at the Johannes Gutenberg University of Mainz Medicine
Langenbeckstr. 1
Building 708
55131 Mainz
Germany

Sequencing libraries

Supplementary Table S1: Sequencing libraries used in this study

Library	Source C57BL/6 mouse individual	RNA/DNA	Read length (nt)
**Black6.1**	1	DNA	1x50
**Black6.2**	2	DNA	1x50
**Black6.3**	3	DNA	1x50
**B16.1**		DNA	1x50
**B16.2**		DNA	1x50
**B16.3**		DNA	1x50
**Black6.1_PE**	1	DNA	2x100
**B16.1_PE**		DNA	2x100
**Black6.1_RNA**	1	RNA	1x50
**Black6.2_RNA**	2	RNA	1x50
**Black6.3_RNA**	3	RNA	1x50

Supplementary Data: Alignment statistics for all samples
(see alignments.xls)

Supplementary Data: Primer sequences
(see primer.xls)

Supplementary Data: Validation results for mutations with an intermediate FDR
(see intermediate_FDR.xls)

Supplementary Data: 12,460 somatic mutations found in triplicate samples
(see somatic_mutations.xls)

Supplementary Method: Selection of a filtering threshold for somatic sniper

For the selection of an appropriate filtering threshold for the reported “somatic score” of SomaticSNiPer, we plotted the frequency of the scores vs. the logarithm of the scores for all possible combinations of datasets. The plots (Supplementary Figure 1) suggest a change of the score distribution at a score around 30; this was selected as cutoff.

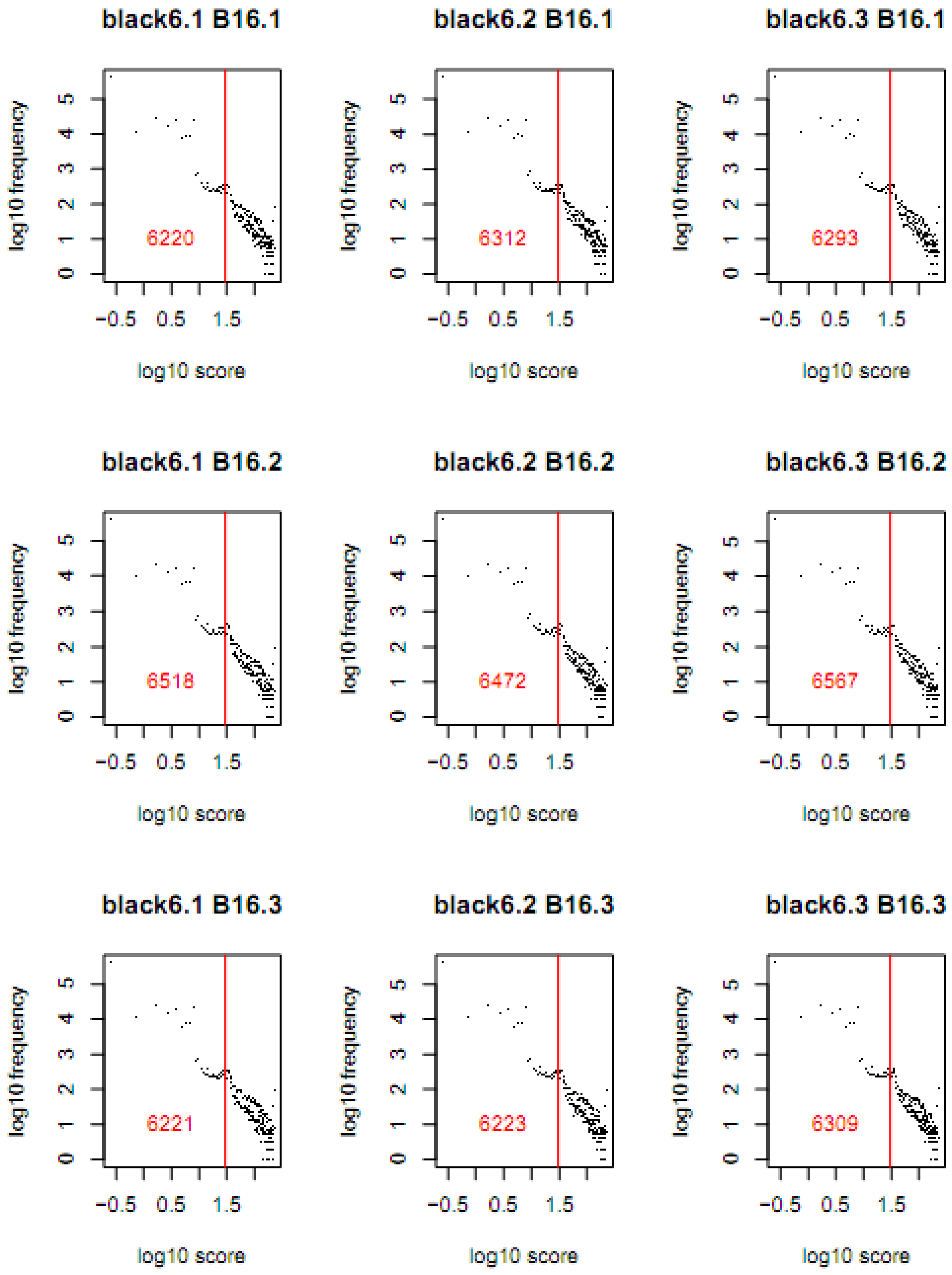

Supplementary Figure S1: log-log plots of somatic score distributions of the SomaticSNiPer results. The red line marks the selected cutoff of 30; the red number gives the number of somatic mutations with a score higher than the cutoff.

Supplementary Results: Complete results of FDR calculation

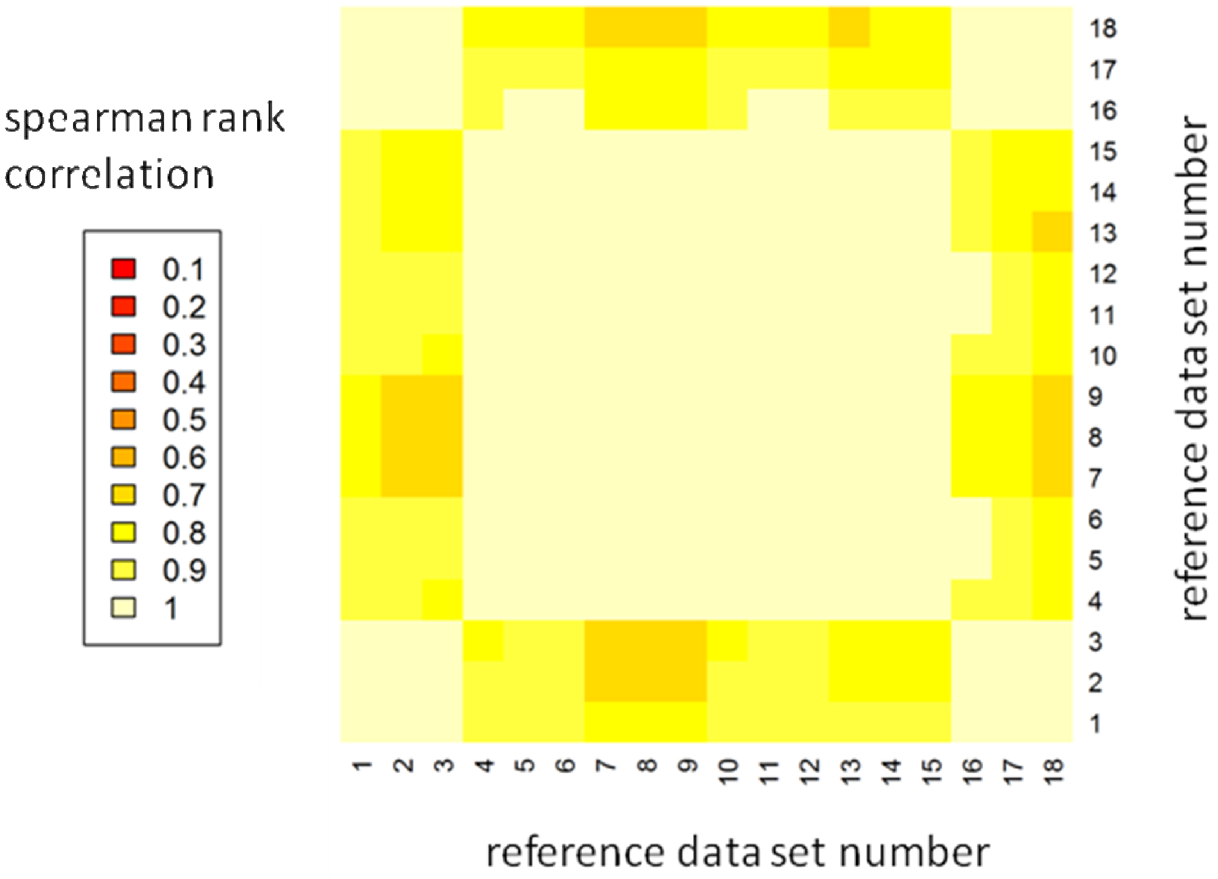

Supplementary Figure S2: Heatmap of the rank correlation between the estimated ROC AUC values for different reference data sets.

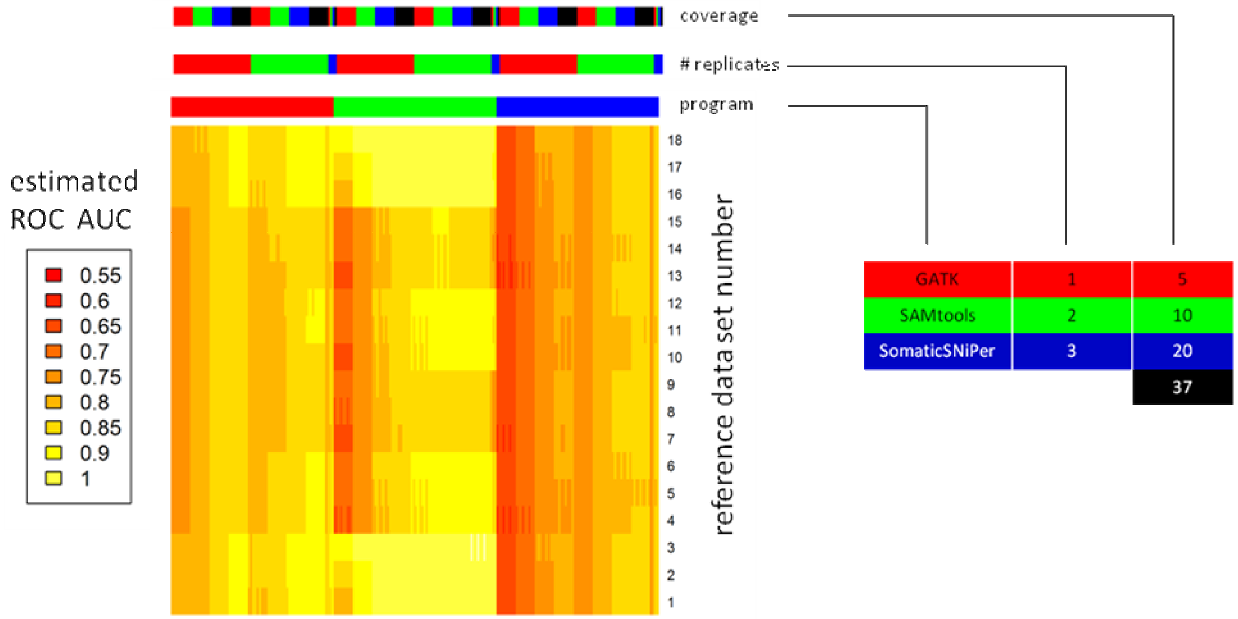

Supplementary Figure S3: Heatmap of the estimated ROC AUC values for all analysis and experimental parameter combinations. The three colored bars at the top encode the respective parameter set for which the AUC values are visualized. In the case of no replicates and one replicates, there are nine possible combinations of the single end libraries, respectively, while for two replicates there is only one combination.

Reference data set numbering

Supplementary Table S2: Numbering of all possible reference set combinations. E.g. the set number 1 includes variations found in library Black6.2 but not in Black6.1 ("same vs. same") and variations found in B16.1 but not in Black6.1 ("same vs. different").

Number	black6 library	black6 library (replicate)	B16 library
**1**	Black6.1	Black6.2	B16.1
**2**	Black6.1	Black6.2	B16.2
**3**	Black6.1	Black6.2	B16.3
**4**	Black6.1	Black6.3	B16.1
**5**	Black6.1	Black6.3	B16.2
**6**	Black6.1	Black6.3	B16.3
**7**	Black6.2	Black6.1	B16.1
**8**	Black6.2	Black6.1	B16.2
**9**	Black6.2	Black6.1	B16.3
**10**	Black6.2	Black6.3	B16.1
**11**	Black6.2	Black6.3	B16.2
**12**	Black6.2	Black6.3	B16.3
**13**	Black6.3	Black6.1	B16.1
**14**	Black6.3	Black6.1	B16.2
**15**	Black6.3	Black6.1	B16.3
**16**	Black6.3	Black6.2	B16.1
**17**	Black6.3	Black6.2	B16.2
**18**	Black6.3	Black6.2	B16.3

Supplementary Discussion: Paired end library results

When deriving different library configurations from the reads of the sequenced 2x100 nt library, we observe an accumulation of somatic mutations in the B16 data with predicted high FDRs (Supplementary Fig. S3). The set of low FDR mutations is rather similar (Supplementary Table 3); note that most potentially somatic mutations are found in the simulated 1x50 nt library. Also the coverage for these accumulated high FDR mutations is rather low (Supplementary Fig. S4).

We investigate the hypothesis that this accumulation of somatic mutations in low coverage regions is an effect of the exome capture procedure; the cDNA fragments are not uniformly distributed but enriched in certain locations (i.e. the target regions defined by the probe sequences). With an fragment length of the original library around 250 nt, both the 2x50 and 1x100 libraries introduce either coverage gaps (being the main difference between the 2x50 and 2x100 libraries as 93.7% of the read pairs align to the same locations, so alignment differences are not prominent) or long read ends sticking out of the enriched regions, also causing low coverage with sequencing errors being misinterpreted as variations (see Supplementary Fig. 5 for an example). Also, the 1x100 nt library obviously has longer reads and higher average coverage than the 1x50 nt library; those are lower quality bases, however, which might confuse both the alignment and mutation call software, possibly explaining the worse performance of the 1x100 nt library compared to the 1x50 nt one. The 2x50 nt 3' library supports this suggestion: this library simulates a ~150 nt fragment length with a small gap between the paired read ends outweighing the worse base quality which is prominent in the 1x50 nt 3' library (producing the worst results).

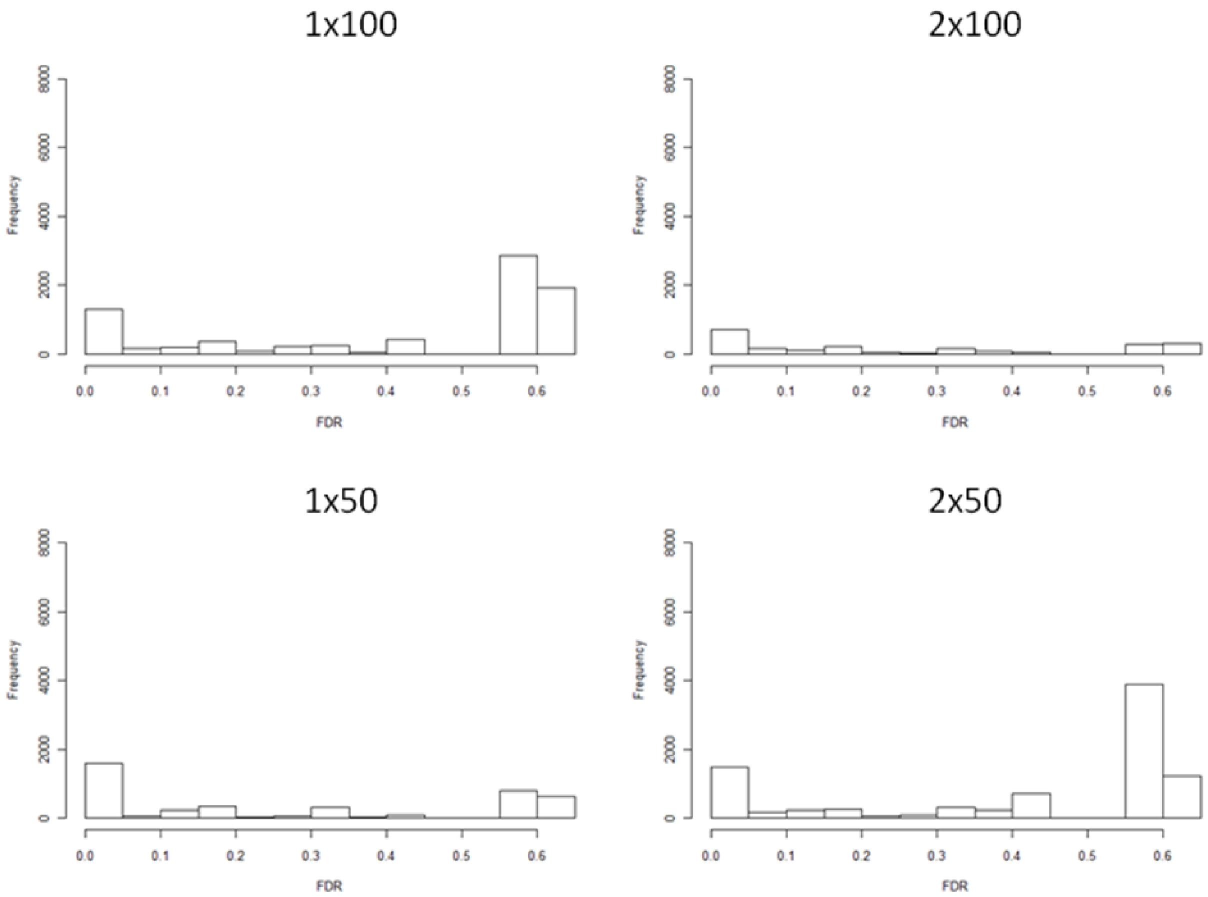

Supplementary Figure S4: FDR distributions for the samtools mutation calls on the aligned reads of the 2x100 nt library (2x100) and the simulated libraries using this data (1x100, 1x50, 2x50).

Supplementary Table S3: Percentage of high quality somatic mutations (FDR < 5%) found in the individual libraries derived from the 2x100 nt B16 library. The total number corresponds to the size of the set of unique somatic mutations (with FDR < 5%) found in all libraries (total number = 2651).

library	% of total mutations found present
**1x50**	85.9
**2x50**	82.6
**1x100**	77.3
**2x100**	65.6

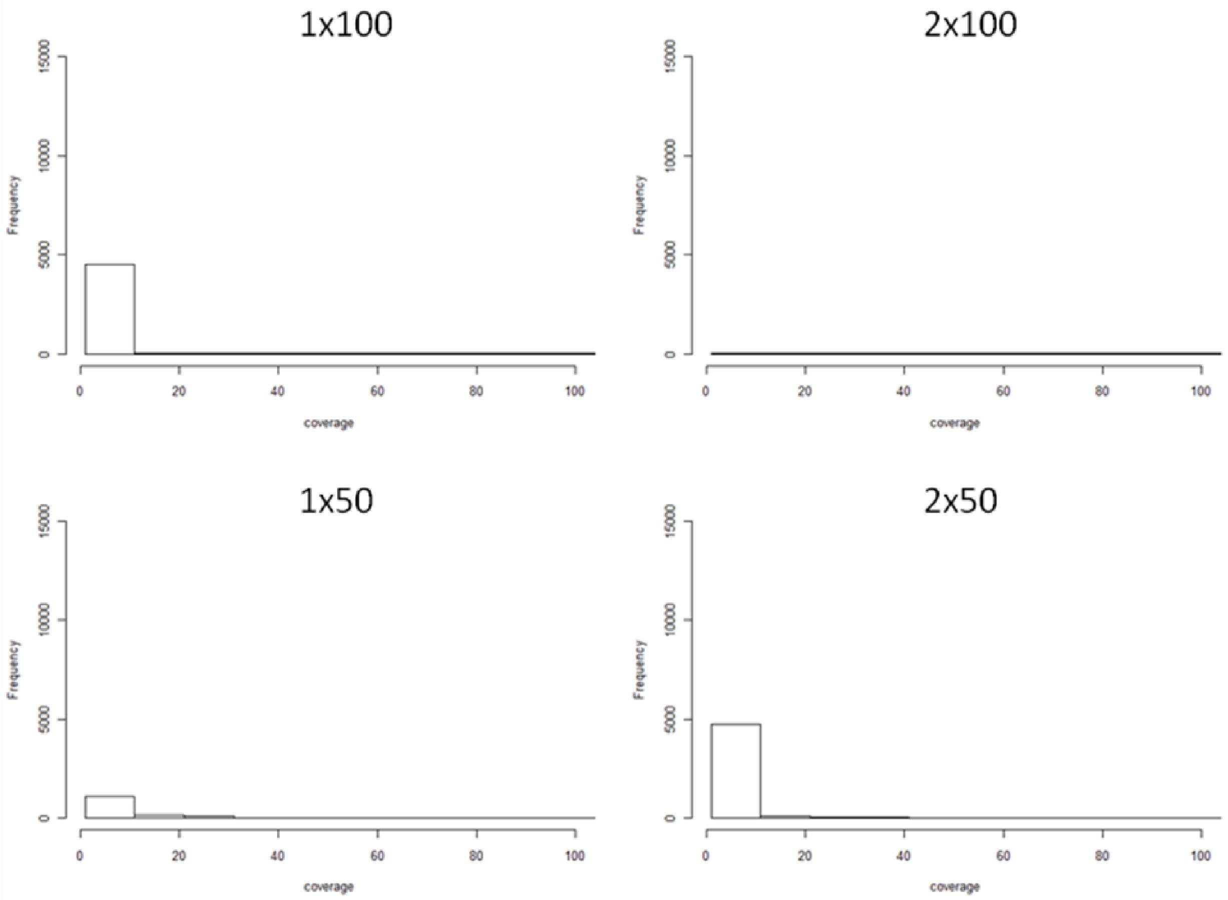

Supplementary Figure S5: Coverage distributions for the samtools mutation calls on the aligned reads of the 2x100 nt library (2x100) and the simulated libraries using this data (1x100, 1x50, 2x50) for all mutations with a FDR higher than 0.5. For better visibility, the x-axes were limited to a maximum value of 100.

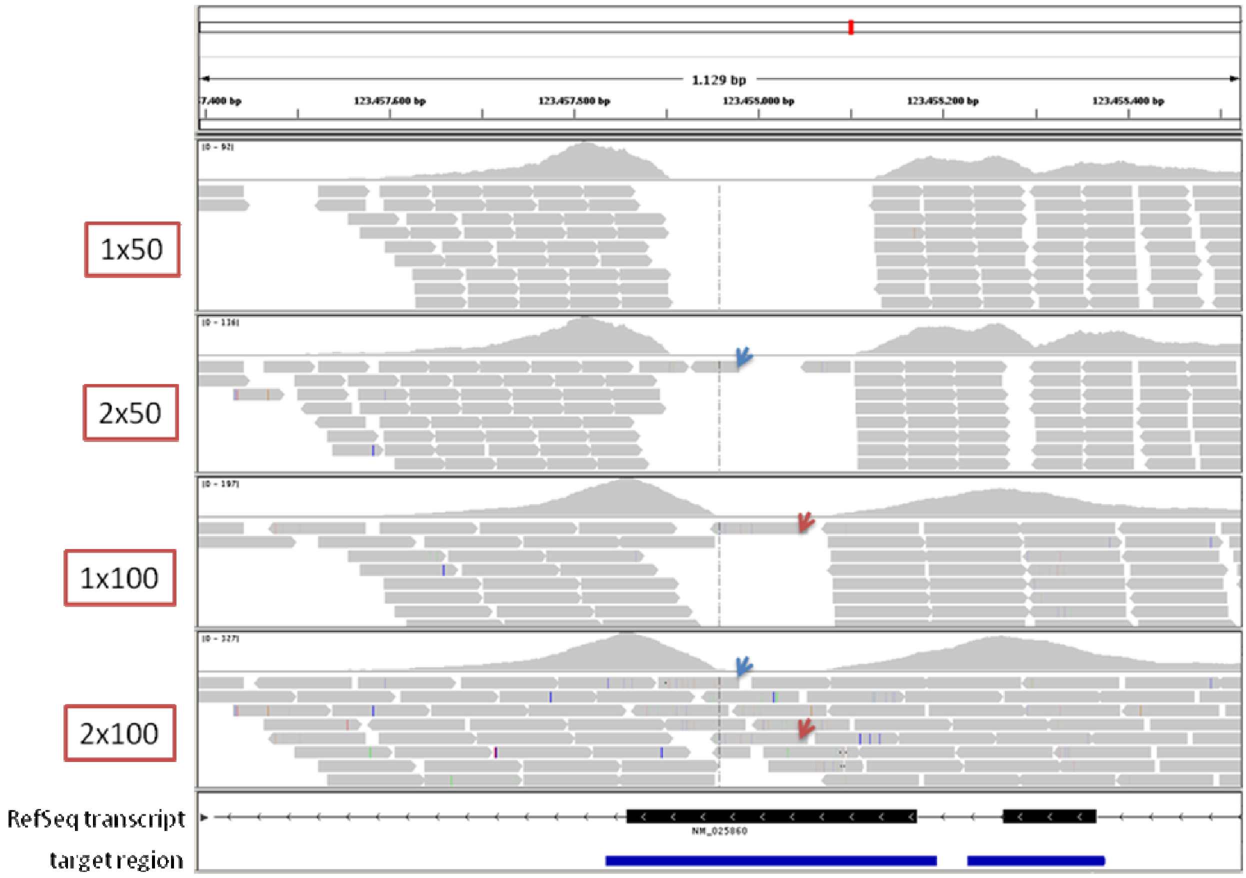

Supplementary Figure S6: Alignments covering a position which is predicted to be a potential somatic mutation in the 2x50 nt and 1x100 nt libraries of B16 but not in the 1x50nt and 2x100 nt libraries. The dashed vertical line marks the locus. The arrows mark two reads; the red and blue arrows in the different panels refer to the same individual read, respectively. Those reads include low quality bases being a possible wrong base call and causing the low quality mutation call. This call is prevented in the 2x100 nt library by higher coverage while in the 1x50 nt case there is simply no coverage.

Effect of replicates

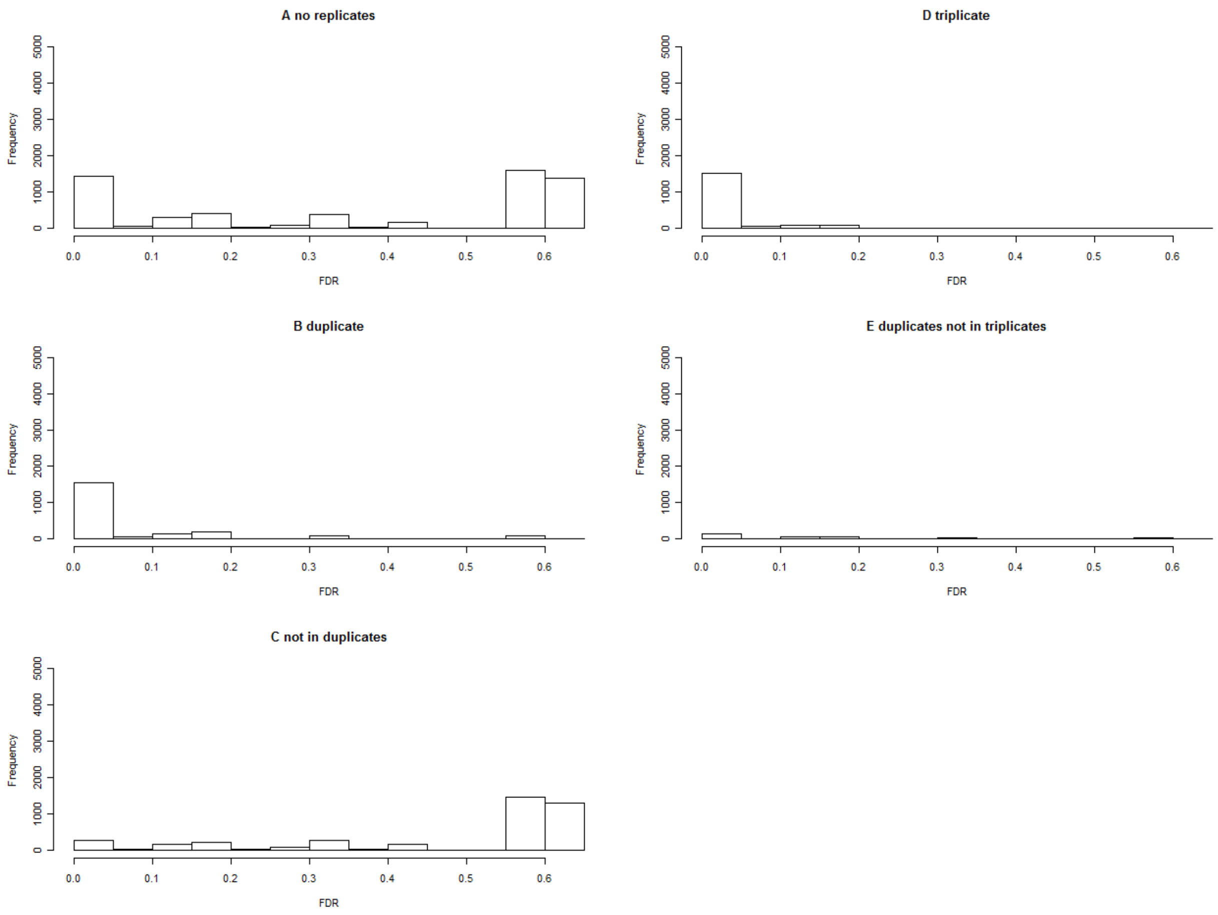

Supplementary Figure S7: FDR distributions for the estimated ROC plots of Figure 4c (A, B, D) and the FDR distributions of the somatic mutations which are removed by the replicate filtering (C, E). The filtering from A to B mostly removes mutations with high FDRs (C), while the filtering using triplicates also removes a comparably large part of the low FDR mutations (E).

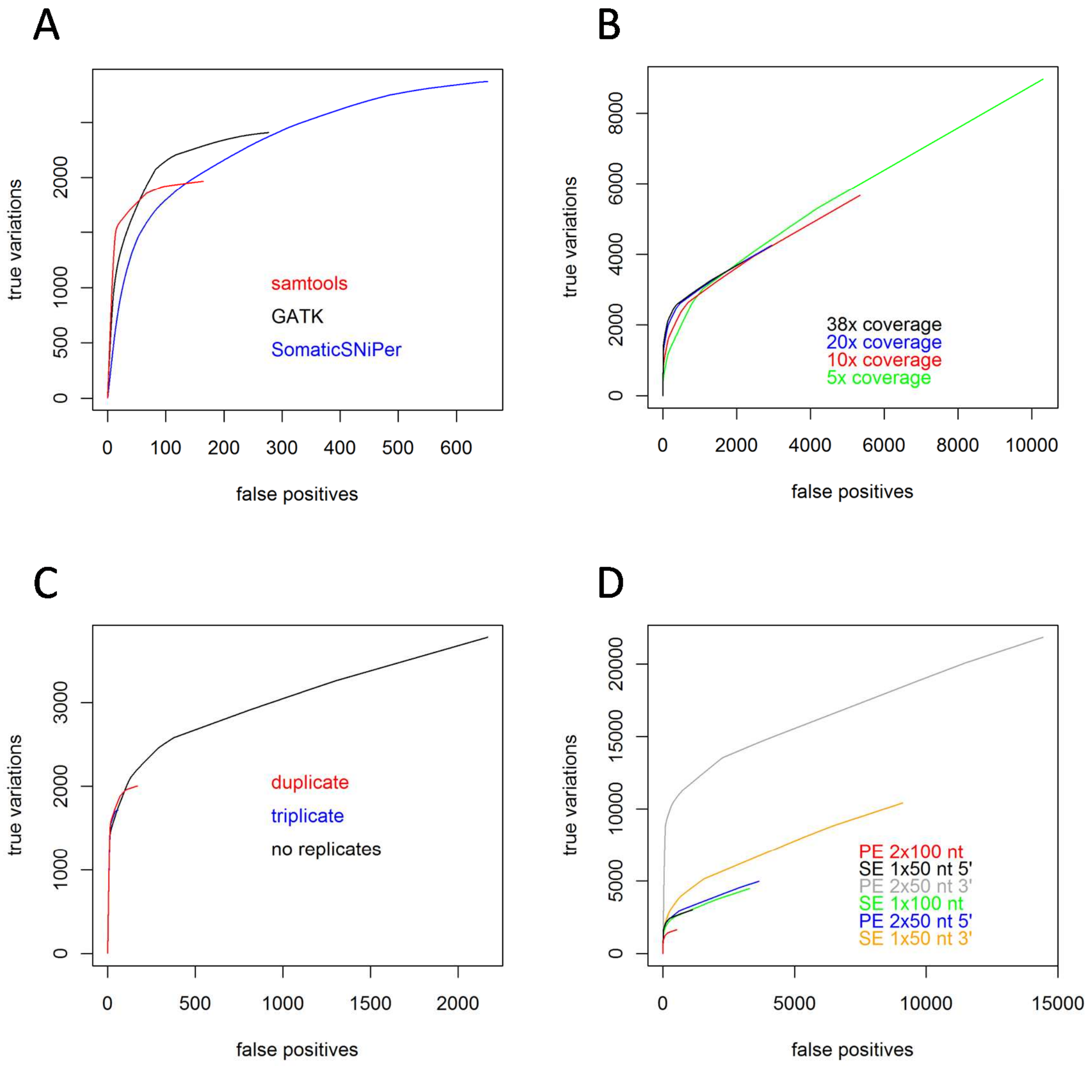

Supplementary Figure S8: (Note: This is an alternative version of Figure 4, skipping the ratio calculation in the ROC curve estimation) **A** Estimated ROC curves for the comparison of the three different software tools (duplicates, 38x coverage). **B** Estimated ROC curves for the comparison of different average sequencing depths (samtools, no replication). 38x denotes the coverage obtained by the experiment, while other coverages were downsampled starting with this data. **C** Estimated ROC curves visualizing the effect of experiment replication (38x coverage, samtools). **D** Estimated ROC curves for different sequencing protocols (samtools, no replication). The curves were calculated using the results of the 2x100 nt library.
